# Determinantes do uso de serviços odontológicos no período pré-gestacional e gestacional: um estudo transversal

**DOI:** 10.1590/0102-311XPT179524

**Published:** 2025-07-21

**Authors:** Letícia Mendes, Bruno Emmanuelli, Maiara de Carvalho Segatto, Jessica Klöckner Knorst, Fernanda Tomazoni, Gabriela Araújo

**Affiliations:** 1 Universidade Federal de Santa Maria, Santa Maria, Brasil.

**Keywords:** Gestantes, Assistência Odontológica, Saúde Bucal, Estudo Observacional, Pregnant People, Dental Care, Oral Health, Observational Study, Personas Embarazadas, Atención Odontológica, Salud Bucal, Estudio Observacional

## Abstract

O serviço odontológico, essencial para a saúde bucal de gestantes, é influenciado por fatores que atuam como barreiras ou facilitadores, impactando mãe e bebê. Este estudo transversal investigou o uso de serviços odontológicos e seus determinantes. Foram avaliadas gestantes usuárias dos serviços públicos de saúde de Santa Maria, Rio Grande do Sul, Brasil, entre abril e outubro de 2022. Questionários registraram dados sociodemográficos, psicossociais e comportamentais. Cárie não tratada e gengivite foram clinicamente avaliadas. O desfecho considerou o uso de serviços odontológicos nos últimos 12 meses e o motivo do uso no último atendimento. Análises de regressão logística multinomial, baseadas no modelo de Andersen & Newman, avaliaram os determinantes do uso de serviços odontológicos. Os resultados são apresentados em razão de chances (OR - *odds ratio*) e respectivos intervalos de 95% de confiança (IC95%). Das 520 gestantes avaliadas, 72,3% utilizaram os serviços. Estar no segundo e terceiro trimestres associou-se ao uso de serviços odontológicos para tratamento no último atendimento (OR = 2,12; IC95%: 1,12-3,74 e OR = 3,92; IC95%: 2,02-6,86, respectivamente). Gestantes solteiras/separadas, com menor escolaridade, baixo senso de coerência e que percebiam barreiras para o uso tiveram, respectivamente, chances 1,63 (IC95%: 1,03-2,56), 2,54 (IC95%: 1,24-5,20), 2,90 (IC95%: 1,25-6,69) e 2,14 (IC95%: 1,10-4,13) vezes maior de não usar os serviços. Assim, demonstrou-se a relação entre determinantes de saúde e o uso de serviços odontológicos no período pré-gestacional e gestacional. Identificar esses aspectos pode contribuir para a reorganização de políticas voltadas a esse grupo.

## Abstract (en)

Dental services, which are essential for the oral health of pregnant women, suffer the influence of factors that either act as barriers or facilitators and impact mothers and babies. This cross-sectional study investigated the use of dental services and its determinants. Pregnant women who used public health services in Santa Maria, Rio Grande do Sul State, Brazil, from April to October 2022 were evaluated. Questionnaires recorded sociodemographic, psychosocial, and behavioral data. Untreated caries and gingivitis were clinically evaluated. The outcome considered the use of dental services in the last 12 months and the reason for use in the last visit. Multinomial logistic regression analyses based on the Andersen & Newman model evaluated the determinants of the use of dental services. Results are shown as odds ratios (OR) and their respective 95% confidence intervals (95%CI). Of the 520 evaluated pregnant women, 72.3% used the services. Being in the second and third trimesters of their gestation was associated with the use of dental services for treatment in their latest visit (OR = 2.12; 95%CI: 1.12-3.74 and OR = 3.92; 95%CI: 2.02-6.86, respectively). Single/separated pregnant women, those with less schooling and low sense of coherence, and those who perceived barriers to use had 1.63 (95%CI: 1.03-2.56), 2.54 (95%CI: 1.24-5.20), 2.90 (95%CI: 1.25-6.69), and 2.14 times (95%CI: 1.10-4.13) higher odds of using no service, respectively. Thus, this study shows the relation between health determinants and the use of dental services in the pre-gestational and gestational periods. Finding these aspects can contribute to the reorganization of policies toward this group.

## Resumen

El servicio odontológico esencial para la salud bucal de las mujeres embarazadas está influenciado por factores que actúan como barreras o facilitadores, lo cual impacta a la madre y al bebé. Este estudio transversal analizó el uso de los servicios odontológicos y sus determinantes. Se evaluó a mujeres embarazadas que utilizaron servicios de salud pública en Santa Maria, Rio Grande do Sul, Brasil, entre abril y octubre de 2022. Los cuestionarios registraron datos sociodemográficos, psicosociales y de comportamiento. La caries y la gingivitis no tratadas se evaluaron clínicamente. El resultado consideró el uso de servicios odontológicos en los últimos 12 meses y la razón del uso en la última visita. Los análisis de regresión logística multinomial con base en el modelo de Andersen & Newman evaluaron los determinantes del uso de los servicios odontológicos. Los resultados se presentan en la razón de probabilidades (OR - *odds ratio*) y respectivos intervalos de 95% de confianza (IC95%). De las 520 mujeres embarazadas evaluadas, el 72,3% utilizó los servicios. Estar en el segundo y tercer trimestre se asoció con el uso de servicios odontológicos para el tratamiento en la última atención (OR = 2,12; IC95%: 1,12-3,74 y OR = 3,92; IC95%: 2,02-6,86, respectivamente). Las mujeres embarazadas solteras/separadas, con menor nivel de estudios, bajo sentido de coherencia y que percibieron barreras de uso tuvieron, respectivamente, 1,63 (IC95%: 1,03-2,56), 2,54 (IC95%: 1,24-5,20), 2,90 (IC95%: 1,25-6,69) y 2,14 (IC95%: 1,10-4,13) probabilidades más altas de no usar los servicios. Así, se demostró la relación entre los determinantes de salud y el uso de servicios odontológicos en el período pregestacional y gestacional. El conocimiento de estos aspectos puede contribuir a la reorganización de las políticas dirigidas a este grupo.

## Introdução

O uso de serviços odontológicos tem sido reconhecido como um determinante da saúde bucal e da qualidade de vida relacionada à saúde bucal de indivíduos desde a infância até a fase de pessoa idosa ^1^. Nesse contexto, identificar o perfil do uso de serviços odontológicos representa um grande desafio para a saúde pública, e tem recebido destaque na literatura em razão do impacto que pode gerar, em especial para grupos de maior vulnerabilidade, como o caso de gestantes ^2^. Aspectos inerentes ao período gestacional, como crenças de pacientes e profissionais em relação à segurança do tratamento odontológico e possíveis mudanças na saúde bucal, podem contribuir para o perfil de uso de serviços odontológicos nesse período ^3^.

No Brasil, o Sistema Único de Saúde (SUS) garante atenção integral à saúde, desde a gestação, visando prevenção e promoção da saúde ^4^. Toda gestante tem direito ao pré-natal completo, incluindo assistência odontológica. O Ministério da Saúde incentiva consultas odontológicas em qualquer fase gestacional, com preferência pelo segundo trimestre, conforme a Cartilha de Saúde Bucal da Gestante ^5^. Segundo a Política Nacional de Saúde Bucal (PNSB), em 2022, 51% das gestantes realizaram pré-natal odontológico, um aumento significativo em relação a 2019 (19%) ^5^. A avaliação do uso de serviços odontológicos pode se dar de várias formas e, comumente, é registrada por meio de questionários ^6^. Quanto ao período considerado na avaliação do uso de serviços odontológicos, uma revisão sistemática recente identificou que ele poderia variar desde sua ocorrência nos últimos seis meses, até alguma visita ao dentista ao longo da vida ^7^. De maneira geral, o uso regular de serviços odontológicos pode ser definido como a realização de uma avaliação odontológica recente ou o uso de serviços odontológicos por razões preventivas ^6^.

No entanto, ainda que a atenção odontológica seja parte importante na prevenção e no tratamento de doenças bucais, e o acesso aos serviços seja considerado universal, seu uso efetivo não é igualmente distribuído entre a população, refletindo desigualdades no acesso e na utilização desses serviços ^6^ ^,^ ^8^. Um dos modelos mais aceitos e utilizados na literatura, o modelo dos determinantes do uso de serviços de saúde de Andersen & Newman ^9^, já apontava o papel de diferentes fatores classificados, originalmente, como aspectos predisponentes, facilitadores e de necessidade, no perfil de uso de serviços odontológicos. A literatura tem evidenciado as disparidades no uso de serviços odontológicos, bem como a associação de diferentes fatores demográficos, socioeconômicos e psicossociais a esse desfecho ^2^ ^,^ ^6^. Nesse sentido, conhecer os determinantes para o uso de serviços odontológicos pode ser útil para a orientação e formulação de intervenções eficazes para ampliar o uso de serviços odontológicos por grupos mais vulneráveis, o que inclui gestantes. Destaca-se o papel fundamental dos determinantes de saúde geral e bucal do futuro binômio mãe-bebê durante o período gestacional, tornando-o um momento oportuno para intervenções e orientações ^10^.

Nesse contexto, o objetivo deste estudo foi investigar o perfil de uso de serviços odontológicos nos últimos 12 meses e seus determinantes, em uma amostra de gestantes atendidas no serviço público de saúde em um município do sul do Brasil. Nossa hipótese conceitual, conforme o modelo para uso de serviços odontológicos proposto por Andersen & Newman ^9^, é de que tanto características predisponentes, quanto facilitadoras e de necessidade estarão associadas ao perfil de uso de serviços odontológicos na amostra avaliada.

## Metodologia

Este estudo foi reportado considerando as recomendações propostas pelo STROBE (*Strengthening the Reporting of Observational Studies in Epidemiology*) ^11^.

### Desenho do estudo e amostra

Esse foi um estudo transversal, em que a coleta de dados ocorreu entre abril e outubro de 2022. A amostra abrangeu gestantes usuárias dos serviços públicos de saúde (unidades básicas de saúde - UBS - e Estratégia Saúde da Família - ESF) do Município de Santa Maria, Rio Grande do Sul, Brasil. Na ocasião do levantamento, a cidade possuía uma população estimada de 285.159 habitantes ^12^ e, aproximadamente, 1.380 gestantes cadastradas em UBSs e ESFs, conforme dados da Secretaria Municipal de Saúde. A seleção da amostra foi realizada em 25 unidades de saúde (UBSs e ESFs) do município, abrangendo as oito regiões administrativas da cidade. Utilizou-se um plano amostral por conglomerados, no qual a alocação das gestantes foi proporcional ao porte de cada unidade. Assim, a proporção de gestantes a serem incluídas em cada unidade considerou o peso amostral, calculado a partir da razão entre o número de gestantes cadastradas na unidade e o total de gestantes atendidas pelo serviço público municipal no início do estudo. Por exemplo, uma unidade com 150 gestantes cadastradas contribuiu com 10,9% (150/1.380 × 100 = 10,9) da amostra total. Dessa forma, garantiu-se que unidades maiores contribuíssem com maior número de participantes. Dentro de cada unidade, a seleção das gestantes foi realizada de forma aleatória por meio de sorteio simples.

Foram consideradas todas as gestantes cadastradas nas UBSs e EFSs do município. Não foram consideradas elegíveis gestantes portadoras de aparelho ortodôntico fixo ou contenção ortodôntica, pela dificuldade de avaliação clínica, bem como gestantes que utilizavam medicamentos associados ao aumento de volume gengival (nifedipina, ciclosporina e fenitoína), ou com sinais claros de comprometimento cognitivo no momento da entrevista que pudesse impedir o fornecimento de respostas aos questionários ou inviabilizasse a execução dos exames clínicos. Gestantes que relataram gravidez de risco também não foram consideradas elegíveis.

Em cada unidade, as gestantes cadastradas eram numeradas em sequência, e o sorteio aleatório era realizado pelo site https://www.random.org/. As unidades informavam à equipe de pesquisa os turnos semanais destinados aos atendimentos regulares às gestantes. Nesses dias, a equipe identificava a presença das gestantes sorteadas, realizando o primeiro contato para verificar a elegibilidade. Caso fossem elegíveis, as gestantes eram convidadas a participar do estudo. Nos casos de recusa, os motivos eram registrados em uma ficha específica com identificação da unidade.

### Cálculo amostral

O cálculo amostral foi realizado por meio do software OpenEpi (http://www.OpenEpi.com) e considerou os seguintes parâmetros: nível de 95% de significância, poder do estudo de 80% para detectar associações, razão entre expostos e não expostos de 1:1, e tamanho de efeito de 0,3 ^13^, que reflete uma magnitude, entre pequena e moderada, de associação esperada entre possíveis preditores e o desfecho, totalizando uma amostra de 352 gestantes. Considerando um efeito do desenho de 1,2 ^14^ e adicionando 20% para compensar possíveis recusas, a amostra mínima requerida foi de 507 gestantes.

### Aspectos éticos

O estudo foi aprovado pelo Comitê de Ética em Pesquisa da Universidade Federal de Santa Maria (protocolo CAAE 54969222.9.0000.5346), com o número do parecer da Plataforma Brasil: 5.229.966. Todas as participantes foram informadas sobre os objetivos da pesquisa e, ao consentirem com a participação, assinaram o Termo de Consentimento Livre e Esclarecido, ou o Termo de Assentimento Livre e Esclarecido para as menores de idade.

### Uso de serviços odontológicos

O desfecho considerado neste estudo foi avaliado por meio de dois aspectos: uso de serviços odontológicos nos últimos 12 meses e o motivo para o uso de serviços no último atendimento. O uso foi avaliado por meio da frequência das consultas ao dentista, sendo adotada a seguinte pergunta validada e utilizada como padrão na *Pesquisa Nacional de Saúde* (PNS) ^15^: “No último ano (12 meses) quantas vezes você foi ao dentista?”. Foram oferecidas as seguintes opções de resposta: “nenhuma vez”, “1 vez”, “2 vezes”, “3 vezes ou mais”. Além do uso de serviços odontológicos, as gestantes foram questionadas quanto ao motivo da última consulta odontológica por meio da pergunta: “Qual o motivo da última consulta?” ^16^, com as seguintes opções de resposta: “dor de dente”, “dor na boca”, “batidas e quedas”, “exame de rotina”, “aparelho”, “outros”, “não foi ao dentista”. Para fins estatísticos, a partir dessas avaliações, foi construída a variável perfil de uso de serviços odontológicos, que apresentou três categorias: (i) uso regular de serviços odontológicos, para prevenção na última consulta (uso de serviço nos últimos 12 meses, com o último atendimento por motivos de prevenção e exames de rotina); (ii) uso para tratamentos na última consulta (uso de serviço odontológico nos últimos 12 meses, com o último atendimento por razões curativas); e (iii) não uso de serviços odontológicos (não uso de serviço odontológico nos últimos 12 meses).

### Avaliação dos possíveis determinantes para o uso de serviços odontológicos

Neste item são descritos os possíveis determinantes para o uso de serviços odontológicos. As variáveis consideradas tiveram como base o modelo conceitual para uso de serviços proposto por Andersen & Newman ^9^. Nesse modelo, o uso de serviços odontológicos está na dependência de três principais aspectos: (i) predisposição, (ii) facilitadores e (iii) necessidade.

#### Predisposição

A predisposição de um indivíduo para o uso de serviços odontológicos está relacionada a seus aspectos socioculturais presentes antes do desenvolvimento de algum tipo de condição de saúde bucal adversa ^9^. Dessa forma, considerou-se as seguintes variáveis: idade, período gestacional, status de relacionamento, educação, cor da pele, ocupação, aglomeração familiar, frequência de prática de atividades religiosas, senso de coerência, letramento em saúde bucal e percepção de barreiras para o uso de serviços odontológicos.

A idade das gestantes foi avaliada em anos e, considerando a relação entre idade e uso de serviços odontológicos apresentada na literatura ^17^ ^,^ ^18^ ^,^ ^19^, foi categorizada em “adolescentes” (13 a 19 anos), “adultas jovens” (20 a 24 anos) e “adultas” (25 a 50 anos). O trimestre gestacional foi registrado em semanas e, posteriormente, categorizado em “primeiro trimestre” (até 12 semanas), “segundo trimestre” (entre 13 e 27 semanas) e “terceiro trimestre” (a partir de 28 semanas). A cor da pele foi coletada conforme critérios estabelecidos pelo Instituto Brasileiro de Geografia e Estatística (IBGE) ^12^ e, posteriormente, dicotomizada em “brancas” ou “pretas e pardas”. A escolaridade foi coletada em anos de ensino formal e dicotomizada em “< 8 anos” e “≥ 8 anos” (correspondente ao Ensino Fundamental no Brasil). A aglomeração familiar foi obtida por meio da divisão do número de pessoas por domicílio pelo número de cômodos na casa (espaços cobertos por teto e limitados por paredes) ^20^ sendo posteriormente dicotomizada em “≤ 1” ou “> 1” pessoa por cômodo.

A prática de atividades religiosas foi avaliada pela pergunta: “Com que frequência você pratica atividades religiosas?”, e dicotomizada em “mais frequente” (todos os dias ou quase todos os dias, uma vez na semana) e “menos frequente” (uma vez ao mês, uma vez a cada três meses, e nunca ou quase nunca) ^21^. Essa variável foi utilizada como um *proxy* do capital social individual, o qual tem relação com a utilização e acesso aos serviços de saúde bucal ^21^. O senso de coerência foi mensurado por meio da versão brasileira e reduzida do *Escala de Senso de Coerência* (*Sense of Coherence Scale* - SOC-13) ^22^ ^,^ ^23^. O senso de coerência é medido em uma escala Likert de 1-4, com escores variando de 13-65, em que maiores escores indicam senso de coerência mais alto ^22^. Para a análise, o senso de coerência foi categorizado utilizando a média (45,3) e ±1 desvio padrão (DP = 7,40) da amostra, conforme literatura prévia ^24^, sendo classificado em “baixo” (valores < 37,9), “moderado” (valores entre 38,0 e 52,6) e “alto” (valores > 52,7). O letramento em saúde bucal foi avaliado por meio do Breald-30, versão brasileira adaptada do *Estimativa Rápida da Alfabetização de Adultos em Odontologia* (*Rapid Estimate of Adult Literacy in Dentistry* - REALD-30) ^25^, no qual a pontuação total varia de 0 (menor letramento) a 30 (maior letramento). Para fins de análise, o escore do quartil inferior “< 23” foi definido como o ponto de corte indicando nível “baixo” de letramento em saúde bucal e “≤ 23” indicou nível “alto” de letramento em saúde bucal ^26^.

As participantes foram questionadas quanto à sua percepção em relação às barreiras para a realização do pré-natal ou consulta odontológica por meio da seguinte pergunta: “Dentre as opções abaixo, quais você considera que seja uma barreira para o uso de serviço odontológico durante a sua gestação?”, com as opções de resposta: “dificuldade de pagar pelo atendimento odontológico”; “não ter um plano de saúde”; “o tratamento odontológico não é seguro durante a gestação”; “não encontro profissionais disponíveis para meu atendimento odontológico nesta unidade de saúde”, “não encontro profissionais disponíveis para atendimento odontológico de gestantes”, “não identifico barreiras para o uso de serviços odontológicos” ^27^, para fins de análise estatística, essa variável foi categorizada em: “percebe algum tipo de barreira” e “não percebe nenhum tipo de barreira”.

#### Facilitadores

Os facilitadores dizem respeito a características que facilitam a obtenção de serviços odontológicos, recursos pessoais, familiares e de comunidade ^9^. Nesse sentido, consideraram-se as seguintes variáveis: renda e disponibilidade de serviço odontológico na unidade de saúde, ou estratégia de saúde a qual a gestante estava adscrita. A renda foi coletada pela renda familiar média do mês anterior e codificada pelo salário mínimo brasileiro em 2022 (1 salário mínimo brasileiro equivale a 200 dólares, aproximadamente). A disponibilidade de serviço odontológico na UBS ou ESF a qual a gestante estava adscrita foi considerada como “sim” e “não”.

#### Necessidade

A necessidade relaciona-se às causas mais imediatas, que podem ser percebidas ou avaliadas, e podem estar relacionadas ao uso de serviços odontológicos ^9^. Neste estudo, foram avaliadas a autopercepção de saúde bucal, cárie não tratada e sangramento gengival. A autopercepção de saúde bucal da gestante foi considerada como “excelente/boa” (excelente, boa) e “regular/ruim” (regular, ruim e péssima) ^28^. As variáveis clínicas cárie não tratada e sangramento gengival foram coletadas nas UBSs e ESFs em cadeiras convencionais, com iluminação artificial, utilizando espelho odontológico plano e sondas periodontais (Índice Periodontal Comunitário - CPI, acrônimo em inglês). O sangramento gengival foi examinado em seis sítios em todos os dentes permanentes (exceto terceiros molares) pelo CPI adaptado, e dicotomizado em “< 10%” e “≥ 10%” ^29^; foi considerada presença de cárie dentária não tratada se a gestante apresentasse, pelo menos, uma lesão não tratada (componente C) do índice CPO-D (Índice de Dentes Cariados, Perdidos e Obturados), segundo critérios da Organização Mundial da Saúde (OMS) ^30^.

### Treinamento e calibração

Previamente a coleta de dados, foi realizado um processo de treinamento para a aplicação dos questionários afim de se padronizar a forma de coleta das informações, evitando-se interferências por parte dos entrevistadores. Em relação ao letramento em saúde bucal, foi realizado um treinamento com base em um estudo anterior ^31^, utilizando áudios de dez mulheres que não participaram do estudo. O processo de treinamento para o índice CPI e treinamento e calibração dos cinco examinadores para a aferição do CPO-D foi realizado seguindo o método descrito pela OMS ^32^, por meio de aulas teóricas e treinamento com imagens clínicas. A calibração propriamente dita contou com a participação de mulheres com idades entre 18-45 anos, que não compuseram a amostra final. Os valores de kappa inter-examinadores variaram de 0,76 a 0,88, enquanto os valores de kappa intra-examinadores variaram de 0,80 a 0,89.

### Análise estatística

Os dados foram analisados por meio do programa Stata 14.0 (https://www.stata.com). Uma análise descritiva das variáveis que compõe os três determinantes principais relacionados ao uso de serviços odontológicos foi realizada e é descrita por meio de números absolutos (n) e frequências relativas (%). Além disso, tabelas de contingência e testes de qui-quadrado foram realizados para descrever o perfil de uso de serviços odontológicos, conforme as categorias das variáveis preditoras.

**Figura 1:**
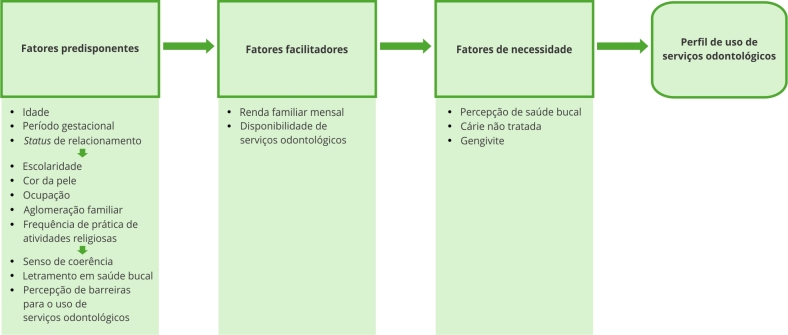
Modelo teórico dos determinantes para a utilização de serviços de saúde, adaptado do modelo originalmente proposto por Andersen & Newman ^9^.

Análise não ajustada de regressão logística multinomial foi, inicialmente, conduzida para verificar possíveis associações entre as variáveis preditoras e o perfil de uso de serviços odontológicos. Na sequência, modelos ajustados de regressão logística multinomial foram construídos. Não se utilizou modelos multiníveis, já que a distribuição do desfecho não mostrou variabilidade contextual. A construção de modelos hierarquizados tomou como base o modelo conceitual para utilização de serviços de saúde proposto por Andersen & Newman ^9^. O modelo adaptado que guiou a construção dos modelos ajustados é apresentado na Figura 1. Cinco modelos são apresentados: (modelos 1, 2 e 3) para predisposição, (modelo 4) para os facilitadores e (modelo 5) para necessidade. No modelo 1, foram incluídas variáveis demográficas, clínicas gestacionais e civis; no modelo 2, foram incluídas variáveis de estrutura social; e, no modelo 3, adicionaram-se variáveis relacionadas a crenças e aspectos individuais. O modelo 4 considerou variáveis facilitadoras. Por fim, o modelo 5 considerou características relacionadas à necessidade. Cada modelo, a partir do modelo 2, foi ajustado pelo(s) seu(s) antecedente(s). Todas as análises foram realizadas considerando-se o peso amostral, por meio do comando *svy* do Stata. Os resultados são apresentados como razões de chance (*odds ratio* - OR) e seus respectivos intervalos de 95% de confiança (IC95%). A qualidade de ajuste dos modelos foi verificada por meio do pseudo R^2^.

## Resultados

Dentre as gestantes sorteadas para compor a amostra (n = 558), duas foram consideradas inelegíveis devido a problemas cognitivos e uma por não ter o Português como língua nativa. Assim, 555 gestantes elegíveis foram convidadas a participar do estudo, das quais 520 aceitaram, resultando em uma taxa de resposta de 93%. Os motivos para recusa incluíram falta de tempo ou desinteresse (n = 29), vergonha ou medo (n = 4) e, em dois casos, diagnóstico de COVID-19, embora essa condição não tenha sido considerada um critério de exclusão. De forma geral, 72% das gestantes utilizaram serviço odontológico no último ano, mas apenas 21,8% referiam-se ao último uso como serviços preventivos (uso regular), enquanto 50,5% tiveram o último uso para tratamento, e 27,7 não utilizaram os serviços odontológicos.

As características gerais da amostra são descritas na Tabela 1. A maioria das gestantes eram adultas (58,1%) e 41,4% estavam no terceiro trimestre de gestação, eram brancas (60%) e Cerca de 71,5% tinham, pelo menos, o Ensino Fundamental completo (71,5%). A maioria das gestantes percebia alguma barreira para o uso de serviços odontológicos (78,7%). Cerca de 37,8% das gestantes ganhavam até 1 salário mínimo, e 70,8% tinham disponibilidade de serviços odontológicos na UBS/ESF em que estavam adscritas. Além disso, 65,4% das gestantes responderam ter uma percepção de saúde bucal regular/ruim, 38,3% apresentaram cárie não tratada e 17,2% apresentaram sangramento gengival.

**Tabela 1:** Características demográficas, socioeconômicas, psicossociais, de saúde bucal e uso de serviços em gestantes atendidas nos serviços públicos de saúde. Santa Maria, Rio Grande do Sul, Brasil (N = 520).

Variáveis	n (%) *
Predisposição **	
Idade	
Adolescentes	66 (13,0)
Adultas jovens	151 (28,9)
Adultas	302 (58,1)
Período gestacional (trimestre)	
Primeiro	108 (21,5)
Segundo	194 (37,1)
Terceiro	212 (41,4)
*Status* de relacionamento	
Casada	163 (31,8)
Solteira/Separada	357 (68,2)
Escolaridade (anos de estudo)	
≥ 8	372 (71,5)
< 8	148 (28,5)
Cor da pele	
Brancas	313 (60,0)
Pretas e pardas	207 (40,0)
Ocupação	
Empregada	229 (44,3)
Não empregada	291 (55,7)
Aglomeração familiar (pessoa por cômodo)	
≤ 1	296 (57,1)
> 1	224 (42,9)
Frequência de prática de atividades religiosas	
Menos frequente	236 (45,0)
Mais frequente	284 (55,0)
Senso de coerência	
Alto	85 (16,2)
Moderado	350 (67,5)
Baixo	85 (16,3)
Letramento em saúde bucal	
Alto	364 (69,8)
Baixo	156 (30,2)
Percepção de barreiras para o uso de serviços odontológicos	
Percebe	387 (78,7)
Não percebe	107 (21,3)
Facilitadoras **	
Renda familiar mensal (salários mínimos brasileiros)	
≤ 1	196 (37.8)
> 1-2	172 (32.8)
> 2	152 (29.4)
Disponibilidade de serviços odontológicos na UBS/ESF	
Sim	372 (70,8)
Não	148 (29,2)
Necessidade **	
Percepção de saúde bucal	
Excelente/Boa	180 (34,6)
Regular/Ruim	340 (65,4)
Cárie não tratada	
Sim	199 (38,3)
Não	321 (61,7)
Gengivite	
Sim	89 (17,2)
Não	431 (82,8)
Desfecho - uso de serviços odontológicos	
Uso regular (para prevenção na última consulta)	110 (21,8)
Uso para tratamento (na última consulta realizada)	263 (50,5)
Não uso	145 (27,7)

ESF: Estratégia Saúde da Família; UBS: unidade básica de saúde.

* Considerando o peso amostral, comando *svy* do software Stata;

** Conforme adaptação do modelo conceitual dos determinantes do uso de serviços de Andersen & Newman ^9^.

A Tabela 2 descreve a prevalência de uso de serviços odontológicos conforme as características demográficas, socioeconômicas, psicossociais e de saúde bucal das gestantes. Período gestacional, *status* de relacionamento, escolaridade, ocupação, aglomeração familiar, frequência de atividades religiosas, senso de coerência, percepção de barreiras para o uso de serviços odontológicos e percepção de saúde bucal demonstraram associação estatisticamente significativa ao perfil de uso de serviços odontológicos (valor de p < 0,05).

**Tabela 2:** Avaliação da prevalência de uso de serviços odontológicos conforme as características demográficas, socioeconômicas, psicossociais e de saúde bucal de gestantes atendidas no serviço público de saúde. Santa Maria, Rio Grande do Sul, Brasil (N = 520).

Variáveis	Perfil de uso de serviços odontológicos			Valor de p ***
	Uso regular *	Uso para tratamento **	Não uso	
	n (%)	n (%)	n (%)	
Predisposição ^#^				
Idade				0,084
Adolescentes	12 (10,9)	28 (11,3)	25 (17,3)	
Adultas jovens	25 (23,0)	89 (33,6)	36 (25,1)	
Adultas	73 (66,2)	145 (55,1)	84 (57,6)	
Período gestacional (trimestre)				< 0,001
Primeiro	33 (32,3)	37 (14,3)	38 (26,5)	
Segundo	41 (37,5)	91 (34,4)	61 (41,7)	
Terceiro	33 (30,2)	134 (51,3)	44 (31,8)	
*Status* de relacionamento				0,046
Casada	42 (38,8)	81 (31,0)	39 (27,4)	
Solteira/Separada	68 (61,2)	182 (69,0)	106 (72,6)	
Escolaridade (anos de estudo)				< 0,001
< 8	20 (18,4)	67 (25,3)	60 (42,1)	
≥ 8	90 (81,6)	196 (74,7)	85 (57,9)	
Cor da pele				0,130
Brancas	65 (58,6)	170 (64,5)	78 (53,7)	
Pretas e pardas	45 (41,4)	93 (35,5)	67 (46,3)	
Ocupação				0,008
Empregada	62 (57,1)	109 (41,9)	58 (39,1)	
Não empregada	48 (42,9)	154 (58,1)	87 (60,9)	
Aglomeração familiar (pessoa por cômodo)				0,008
≤ 1	70 (63,2)	160 (61,1)	64 (44,5)	
> 1	40 (36,8)	103 (38,9)	81 (55,5)	
Frequência de prática de atividades religiosas				0,030
Menos frequente	54 (50,8)	136 (51,8)	94 (64,8)	
Mais frequente	56 (49,2)	127 (48,2)	51 (35,2)	
Senso de coerência				0,043
Alto	20 (17,9)	51 (19,2)	14 (9,5)	
Moderado	75 (68,2)	177 (67,3)	97 (67,4)	
Baixo	15 (13,8)	35 (13,5)	34 (23,1)	
Letramento em saúde bucal				0,494
Alto	82 (73,1)	186 (71,1)	96 (65,8)	
Baixo	28 (26,9)	77 (28,9)	49 (34,2)	
Percepção de barreiras para o uso de serviços odontológicos				0,030
Percebe	76 (72,2)	191 (76,9)	118 (87,2)	
Não percebe	31 (27,8)	58 (23,1)	18 (12,8)	
Facilitadoras ^#^				
Renda familiar mensal (salários mínimos brasileiros)				0,241
≤ 1	33 (30,3)	104 (39,4)	58 (40,5)	
> 1-2	39 (35,6)	84 (31,4)	48 (33,0)	
> 2	38 (34,1)	75 (29,2)	39 (26,5)	
Disponibilidade de serviços odontológicos na UBS/ESF				0,074
Sim	77 (69,2)	200 (75,4)	94 (64,1)	
Não	33 (30,8)	63 (24,6)	51 (35,9)	
Necessidade ^#^				
Percepção de saúde bucal				0,017
Boa	45 (41,4)	101 (38,0)	33 (22,9)	
Regular/Ruim	65 (58,6)	161 (62,0)	112 (77,1)	
Cárie não tratada				0,074
Sim	33 (30,5)	98 (37,6)	67 (45,5)	
Não	77 (69,5)	165 (62,4)	78 (54,5)	
Gengivite				0,367
Sim	15 (14,0)	43 (16,2)	30 (21,0)	
Não	95 (86,0)	220 (83,8)	115 (79,0)	

ESF: Estratégia Saúde da Família; UBS: unidade básica de saúde.

* Uso para prevenção na última consulta realizada;

** Uso para tratamento na última consulta realizada;

*** Valores do teste de qui-quadrado; considerando o peso amostral, comando *svy* do Stata;

^#^ Conforme adaptação do modelo conceitual dos determinantes do uso de serviços de Andersen & Newman ^9^.

A análise não ajustada entre as variáveis de predisposição, facilitadoras e de necessidade e o uso de serviços odontológicos é descrita na Tabela 3. Gestantes que estavam no segundo e terceiro trimestres de gestação, assim como aquelas não empregadas, apresentaram maior chance de referir uso de serviço odontológico para tratamento na última consulta realizada. Gestantes solteiras/separadas, com menor escolaridade, residentes em locais com maior aglomeração familiar, com baixo senso de coerência, que percebiam barreira para o uso de serviços odontológicos, que apresentavam percepção de saúde bucal regular/ruim e com cárie não tratada tiveram maiores chances de não utilizar o serviço odontológico. Em contrapartida, gestantes com maior frequência em atividades religiosas e renda > 2 salários mínimos tiveram menor chance de não usar serviços odontológicos.

**Tabela 3:** Associação não ajustada entre as variáveis de predisposição, facilitadoras e de necessidade e o uso de serviços odontológicos, determinada por meio de regressão logística multinomial.

Variáveis	Perfil de uso de serviços odontológicos		
	Uso regular * **	Uso para tratamento *** ^#^	Não uso ^#^
	OR (IC95%)	OR (IC95%)	OR (IC95%)
Predisposição ^##^			
Idade			
Adolescentes	1,00	1,00	1,00
Adultas jovens		1,41 (0,60-3,27)	0,68 (0,32-1,45)
Adultas		0,80 (0,36-1,78)	0,54 (0,22-1,31)
Período gestacional (trimestre)			
Primeiro	1,00	1,00	1,00
Segundo		2,06 (1,12-3,79)	1,36 (0,74-2,47)
Terceiro		3,82 (1,90-7,67)	1,29 (0,63-2,61)
*Status* de relacionamento			
Casada	1,00	1,00	1,00
Solteira/Separada		1,40 (0,93-2,11)	1,68 (1,10-2,56)
Escolaridade (anos de estudo)			
≥ 8		1,00	1,00
< 8		1,50 (0,79-2,84)	3,20 (1,79-5,82)
Cor da pele			
Brancas	1,00	1,00	1,00
Pretas e pardas		0,77 (0,50-1,22)	1,21 (0,65-2,27)
Ocupação			
Empregada	1,00	1,00	1,00
Não empregada		1,85 (1,26-2,69)	2.07 (1,28-3,35)
Aglomeração familiar (pessoa por cômodo)			
≤ 1	1,00	1,00	1,00
> 1		1,09 (0,75-1,58)	2,14 (1,14-4,02)
Frequência de prática de atividades religiosas			
Menos frequente	1,00	1,00	1,00
Mais frequente		0,95 (0,62-1,46)	0,56 (0,36-0,85)
Senso de coerência			
Alto	1,00	1,00	1,00
Moderado		0,92 (0,46-1,82)	1,86 (0,80-4,28)
Baixo		0,90 (0,52-1,55)	3,13 (1,46-6,72)
Letramento em saúde bucal			
Alto	1,00	1,00	1,00
Baixo		1,10 (0,62-1,95)	1,41 (0,76-2,50)
Percepção de barreiras para o uso de serviços odontológicos			
Percebe	1,00	1,28 (0,83-1,98)	2,62 (1,39-4,94)
Não percebe		1,00	1,00
Facilitadores ^##^			
Renda familiar mensal (salários mínimos brasileiros)			
≤ 1	1,00	1,00	1,00
> 1-2		0,67 (0,45-1,01)	0,69 (0,38-1,22)
> 2		0,65 (0,39-1,22)	0,58 (0,34-0,98)
Disponibilidade de serviços odontológicos na UBS/ESF			
Sim	1,00	1,00	1,00
Não		0,73 (0,46-1,17)	1,26 (0,74-2,15)
Necessidade ^##^			
Percepção de saúde bucal			
Excelente/Boa	1,00	1,00	1,00
Regular/Ruim		1,15 (0,69-1,93)	2,38 (1,32-4,30)
Cárie não tratada			
Sim	1,00	1,37 (0,77-2,23)	1,90 (1,01-3,55)
Não		1,00	1,00
Gengivite			
Sim	1,00	1,19 (0,57-2,48)	1,64 (0,93-2,89)
Não		1,00	1,00

ESF: Estratégia Saúde da Família; IC95%: intervalo de 95% de confiança; OR: *odds ratio*; UBS: unidade básica de saúde.

* Uso para prevenção na última consulta realizada;

** Categoria de referência para a variável uso de serviços odontológicos;

*** Uso para tratamento na última consulta realizada;

^#^ Considerando o peso amostral, comando *svy* do Stata;

^##^ Conforme adaptação do modelo conceitual dos determinantes do uso de serviços de Andersen & Newman ^9^.

A Tabela 4 descreve os resultados da análise hierarquizada entre as variáveis de predisposição, facilitadores e necessidade com o uso de serviços odontológicos pelas gestantes. Considerando as variáveis de predisposição, o período gestacional esteve associado ao uso para tratamento (na última consulta), gestantes no segundo e no terceiro trimestres tiveram, respectivamente, chances 2,12 (IC95%: 1,12-3,74) e 3,92 (IC95%: 2,02-6,86) vezes maior de referir uso de serviço odontológico para tratamento na última consulta realizada. Além disso, gestantes solteiras/separadas, com menor escolaridade, com baixo senso de coerência, assim como aquelas que percebiam barreiras para o uso de serviços odontológicos, tiveram, respectivamente, chances 1,63 (IC95%: 1,03-2,56), 2,54 (IC95%: 1,24-5,20), 2,90 (IC95%: 1,25-6,69) e 2,14 (IC95%: 1,10-4,13) vezes maior de não usar os serviços odontológicos. Gestantes com maior frequência de prática de atividades religiosas mostraram menor chance de não usar o serviço odontológico (OR = 0,51; IC95%: 0,30-0,87). As variáveis facilitadoras (renda familiar e presença de dentista na UBS/ESF) não foram significativamente associadas ao perfil de uso dos serviços odontológicos. As necessidades percebidas e avaliadas clinicamente também não foram associadas ao desfecho no modelo hierarquizado final.

**Tabela 4:** Associação ajustada entre as variáveis de predisposição, facilitadores e necessidade com o uso de serviços odontológicos por gestantes, determinado por meio de regressão logística multinomial.

Variáveis	Perfil de uso de serviços odontológicos		
	Uso regular * **	Uso para tratamento *** ^#^	Não uso ^#^
	OR (IC95%)	OR (IC95%)	OR (IC95%)
Predisposição ^##^			
Modelo 1 - variáveis sociodemográficas			
Idade			
Adolescentes	1,00	1,00	1,00
Adultas jovens		1,57 (0,63-3,91)	0,72 (0,32-1,61)
Adultas		1,02 (0,41-2,52)	0,67 (0,25-1,80)
Período gestacional (trimestre)			
Primeiro	1,00	1,00	1,00
Segundo		2,12 (1,15-3,90)	1,32 (0,73-2,39)
Terceiro		3,92 (2,02-7,65)	1,26 (0,63-2,52)
*Status* de relacionamento			
Casada	1,00	1,00	1,00
Solteira/Separada		1,41 (0,90-2,21)	1,63 (1,03-2,56)
Modelo 2 - variáveis de estrutura social			
Escolaridade (anos de estudo)			
≥ 8	1,00	1,00	1,00
< 8		1,23 (0,61-2,49)	2,54 (1,24-5,20)
Cor da pele			
Brancas	1,00	1,00	1,00
Pretas e pardas		0,81 (0,48-1,36)	1,18 (0,59-2,37)
Ocupação			
Empregada	1,00	1,00	1,00
Não empregada		1,46 (0,87-2,46)	1,47 (0,84-2,57)
Aglomeração familiar (pessoa por cômodo)			
≤ 1	1,00	1,00	1,00
> 1		1,11 (0,73-1,71)	1,80 (0,95-3,43)
Frequência de prática de atividades religiosas			
Menos frequente	1,00	1,00	1,00
Mais frequente		0,99 (0,63-1,55)	0,51 (0,30-0,87)
Modelo 3 - crenças			
Senso de coerência			
Alto	1,00	11,00	1,00
Moderado		0,87 (0,44-1,72)	1,91 (0,66-5,49)
Baixo		0,94 (0,52-1,71)	2,90 (1,25-6,69)
Letramento em saúde bucal			
Alto	1,00	1,00	1,00
Baixo		1,12 (0,64-1,97)	1,02 (0,52-2,02)
Percepção de barreiras para o uso de serviços odontológicos			
Não percebe	1,00	1,00	1,00
Percebe		1,16 (0,73-1,85)	2,14 (1,10-4,13)
Facilitadores ^##^			
Modelo 4			
Renda familiar mensal (salários mínimos brasileiros)			
≤ 1	1,00	1,00	1,00
> 1-2		0,89 (0,49-1,63)	1,09 (0,55-2,14)
> 2		0,96 (0,43-2,12)	1,25 (0,72-2,16)
Disponibilidade de serviços odontológicos na UBS/ESF			
Sim	1,00	1,00	1,00
Não		0,78 (0,44-1,38)	1,43 (0,73-2,78)
Necessidade ^##^			
Modelo 5			
Percepção de saúde bucal			
Boa	1,00	1,00	1,00
Regular/Ruim		1,05 (0,55-2,02)	1,77 (0,87-3,62)
Cárie não tratada			
Não	1,00	1,00	1,00
Sim		1,50 (0,85-2,62)	1,62 (0,78-3,37)
Gengivite			
Não	1,00	1,00	1,00
Sim		0,97 (0,44-2,14)	1,67 (0,89-3,13)

ESF: Estratégia Saúde da Família; IC95%: intervalo de 95% de confiança; OR: *odds ratio*; UBS: unidade básica de saúde.

* Uso para prevenção na última consulta realizada;

** Categoria de referência;

*** Uso para tratamento na última consulta realizada;

^#^ Considerando o peso amostral, comando *svy* do Stata;

^##^ Conforme adaptação do modelo conceitual dos determinantes do uso de serviços de Andersen & Newman ^9^.

## Discussão

Este estudo avaliou o perfil e os determinantes sociais associados ao uso de serviços odontológicos, nos últimos 12 meses, por gestantes atendidas no serviço público de saúde. Observou-se baixo uso regular desses serviços. Além disso, conforme o modelo proposto por Andersen & Newman ^9^, apenas fatores predisponentes estiveram associados ao perfil de uso. Esses achados corroboram parcialmente a hipótese conceitual previamente apresentada, que previa a associação dos três domínios do modelo, isto é, características predisponentes, facilitadoras e de necessidade ao perfil de uso de serviços odontológicos.

Estudos em diferentes países indicam baixa procura por serviços odontológicos durante a gravidez ^33^ ^,^ ^34^ ^,^ ^35^ ^,^ ^36^, com prevalências variando de 23,7% a 50%. No Brasil, segundo o último levantamento nacional de saúde bucal ^37^, 43% dos adultos (35-44 anos) usaram serviços odontológicos nos últimos 12 meses. Entre as gestantes avaliadas no período da coleta, a prevalência de uso foi de 72%. No entanto, 50,5% recorreram ao serviço por razões curativas na última consulta, o que, apesar de limitações discutidas adiante, pode indicar uso preventivo restrito entre mulheres adscritas ao serviço público no período pré-gestacional e gestacional.

A baixa procura por serviços odontológicos para prevenção ou sua não utilização pode estar relacionada a barreiras como desafios domésticos, compromissos de trabalho, restrições financeiras, atitudes adversas de profissionais, medo, ansiedade, insegurança sobre procedimentos odontológicos na gravidez, além de crenças e tabus sobre o cuidado odontológico gestacional ^27^ ^,^ ^38^ ^,^ ^39^ ^,^ ^40^. Como não foi possível determinar se o tratamento ocorreu durante a gestação, não se pode afirmar que esses fatores influenciaram esse comportamento. No entanto, gestantes no segundo e terceiro trimestres relataram maior uso curativo na última consulta. Outro fator associado ao uso de serviços odontológicos é o senso de coerência. A literatura sugere que eventos atípicos, como a gestação, podem influenciá-lo, tornando as mulheres mais flexíveis na tomada de decisões sobre saúde e mais conscientes de seus recursos internos quando possuem senso de coerência elevado ^41^. Nossos achados mostraram que participantes com baixo senso de coerência não utilizaram os serviços odontológicos nos últimos 12 meses. Em contrapartida, maior frequência de atividades religiosas reduziu a chance de não uso, funcionando como capital social, ampliando redes de apoio, promovendo confiança e senso de pertencimento, o que pode influenciar positivamente comportamentos de saúde bucal devido à pressão dos pares ^42^.

Baixa escolaridade foi associada ao não uso de serviços odontológicos, achado também relatado por Kozen et al. ^43^. Essa variável pode representar o nível socioeconômico das participantes, influenciando oportunidades sociais e decisões em saúde. A maior escolaridade, além de favorecer um bom emprego e melhores condições de renda e moradia ^44^, também pode refletir prestígio, acesso a informações e influência social, determinando escolhas e restrições em saúde ^45^ ^,^ ^46^. Assim, melhor posição socioeconômica poderia ampliar o acesso e uso de serviços de saúde e influenciar decisões sobre a própria saúde ^43^. Gestantes solteiras também apresentaram maior chance de não utilizar serviços odontológicos, possivelmente devido a fatores socioeconômicos e psicossociais, como menor suporte financeiro e social, que impactam o acesso e a prioridade dada ao cuidado com a saúde. O apoio do parceiro pode favorecer a adoção de comportamentos preventivos ^47^ ^,^ ^48^ ^,^ ^49^.

A percepção de barreiras pelas gestantes foi associada ao não uso de serviços odontológicos. Essa associação poderia ser ainda maior caso o desfecho considerasse apenas o período gestacional, pois algumas participantes podem ter utilizado o serviço no período pré-gestacional. Estudos realizados no Brasil ^50^ ^,^ ^51^ identificaram como principais barreiras crenças populares que desencorajam o atendimento odontológico, baixa percepção de necessidade e medo da dor. Além disso, uma revisão narrativa apontou a falta de informação, dificuldades de mobilidade e segurança, barreiras financeiras, tempo e insegurança dos profissionais quanto a tratamentos, medicamentos e exames ^52^.

Embora as UBS/ESF disponham de dentistas, incentivos e informações sobre consultas odontológicas, muitas mulheres, tanto no período pré-gestacional quanto gestacional, não utilizam os serviços. O não uso pode estar relacionado a dúvidas sobre os procedimentos e crenças equivocadas, como a ideia de que a gestação predispõe a doenças periodontais ^53^. Apesar de 72% das gestantes terem utilizado o serviço nos últimos 12 meses, a prevalência de cárie não tratada foi alta (38,3%), sendo maior entre aquelas que não o utilizaram (45,5%). Entre as que usaram o serviço e ainda apresentaram cárie não tratada, possíveis explicações incluem prestação irregular do atendimento, medo, ansiedade e estigma sobre o tratamento odontológico na gestação ^54^. É importante diferenciar acesso e uso dos serviços. O acesso refere-se à possibilidade de obter atendimento, considerando disponibilidade profissional, localização, tempo de espera e barreiras financeiras e organizacionais, enquanto o uso corresponde à efetiva realização de consultas e tratamentos ^8^ ^,^ ^26^ ^,^ ^27^. Assim, as barreiras identificadas afetam o acesso, mas também podem impactar o uso dos serviços.

Os resultados devem ser interpretados com cautela, pois nossos achados são aplicáveis apenas a gestantes usuárias do SUS na atenção primária, não representando aquelas atendidas por planos de saúde ou no setor privado. No entanto, o estudo visou especificamente esse grupo, utilizando estratégias metodológicas que garantiram uma amostra representativa. O objetivo principal foi avaliar o perfil de uso de serviços odontológicos entre gestantes, e não exclusivamente durante a gravidez. Reconhecemos a importância do cuidado pré-gestacional, dada sua relação com condições de saúde que podem afetar a saúde materna e fetal ^55^. No entanto, incluir esse período na avaliação pode ter limitado os achados, como na relação entre percepção de barreiras e não uso dos serviços durante a gestação, que pode ter sido atenuada pela forma de aferição do desfecho.

Uma limitação do estudo é que o motivo do uso dos serviços considerou apenas a última consulta, e não os 12 meses anteriores à coleta de dados, o que pode ter subestimado o uso por motivos preventivos, uma vez que a gestante pode ter realizado esse tipo de consulta ao longo do período e recebido tratamento apenas na última visita. No entanto, essa abordagem é consistente com a literatura prévia ^56^. Além disso, a renda não foi associada ao desfecho, possivelmente devido ao ajuste por outros fatores no modelo de Andersen & Newman ^9^. Isso pode refletir interações entre variáveis ou a complexidade do papel do *status* socioeconômico na utilização dos serviços. A ausência de associação também pode estar relacionada a vieses de resposta ou à forma de mensuração da renda, que não foi *per capita*. No entanto, esse método é respaldado pela literatura ^46^, além do fato de termos outros indicadores socioeconômicos ^57^.

Nosso estudo apresenta limitações inerentes a um delineamento transversal, impossibilitando a análise de causalidade e a mensuração do tempo de exposição a determinados fatores. Além disso, alguns aspectos do modelo de Andersen & Newman ^9^ não foram contemplados, podendo haver outras características associadas ao uso de serviços odontológicos nesse grupo. Procuramos utilizar determinantes que têm apresentado associações consistentes, na literatura, ao uso de serviços odontológicos, quando investigados em outros grupos populacionais Estudos futuros devem explorar determinantes estruturais e organizacionais do sistema de saúde, impactos de políticas públicas e aspectos culturais e de crenças sobre cuidados odontológicos.

Apesar das limitações, este estudo apresenta pontos positivos. A análise do uso de serviços odontológicos em gestantes, um período crítico da vida da mulher, é relevante, especialmente considerando que o SUS prioriza esse grupo e promove programas de educação em saúde bucal ^5^. Além disso, a amostra representativa de gestantes atendidas em UBSs e ESFs, distribuídas uniformemente pelas regiões do município, possibilitou uma avaliação abrangente do perfil de uso dos serviços. Esses achados podem embasar estratégias de saúde pública para ampliar a utilização regular desses serviços.

Este estudo identificou baixa prevalência do uso de serviços odontológicos para prevenção e alta para tratamento, além de elevada taxa de não uso entre gestantes atendidas no SUS. Gestantes no segundo e terceiro trimestres tiveram maior chance de relatar consulta odontológica para tratamento. A prática frequente de atividades religiosas reduziu a chance de não uso de serviços odontológicos, enquanto ser solteira/separada, ter baixo senso de coerência, baixa escolaridade e perceber barreiras ao atendimento aumentaram essa chance, evidenciando a influência de fatores socioeconômicos e psicossociais.
